# General Anesthetic Care of Obese Patients Undergoing Surgery: A Review of Current Anesthetic Considerations and Recent Advances

**DOI:** 10.7759/cureus.41565

**Published:** 2023-07-08

**Authors:** Zahra Waheed, Faiza Amatul-Hadi, Amritpal Kooner, Muhammad Afzal, Rahma Ahmed, Harshawardhan Pande, Moses Alfaro, Amber Lee, Joravar Bhatti

**Affiliations:** 1 Anesthesia, Shaukat Khanum Memorial Cancer Hospital and Research Centre, Lahore, PAK; 2 Medical School, Mercer University School of Medicine, Macon, USA; 3 Medical School, Midwestern University Chicago College of Osteopathic Medicine, Downers Grove, USA; 4 Medical School, St. George's University School of Medicine, True Blue, GRD; 5 Medical School, Kennesaw State University, Kennesaw, USA; 6 Medical School, Saint Louis University, St. Louis, USA; 7 Medical School, Long School of Medicine at University of Texas Health Science Center San Antonio, San Antonio, USA; 8 Medical School, Arkansas College of Osteopathic Medicine, Fort Smith, USA

**Keywords:** body mass index, anesthetic management, surgery, anesthesia, obesity

## Abstract

Obesity has long been linked to adverse health effects over time. As the prevalence of obesity continues to rise, it is important to anticipate and minimize the complications that obesity brings in the anesthesia setting during surgery. Anesthetic departments must recognize the innumerable risks when managing patients with obesity undergoing surgery, including anatomical and physiological changes as well as comorbidities such as diabetes, cardiovascular diseases, and malignancies.

Therefore, the purpose of this review is to analyze the current literature and evaluate the current and recent advances in anesthetic care of obese patients undergoing surgery, to better understand the specific challenges this patient population faces. A greater understanding of the differences between anesthetic care for obese patients can help to improve patient care and the specificity of treatment. The examination of the literature will focus on differing patient outcomes and safety precautions in obese patients as compared to the general population. Specifically highlighting the differences in pre-operative, intra-operative, and post-operative care, with the aim to identify issues and present possible solutions.

## Introduction and background

Obesity is defined by the World Health Organization (WHO) as excessive or abnormal fat accumulation that poses negative health risks to patients; to be considered obese, patients must have a body mass index (BMI) of over 30 kg/m^2^ [1]. The rates of obesity have nearly tripled since 1975 representing a growing health concern for healthcare systems worldwide [1]. The 2017-2018 National Health and Nutrition Examination Survey (NHANES) reports 43.4% of adults were identified as obese in the United States alone [2]. The prevalence of obesity is associated with several comorbid conditions, including dyslipidemia, type 2 diabetes, hypertension, and coronary heart disease [3]. It is also associated with various increased health risks including stroke, gallbladder disease, respiratory problems, sleep apnea, osteoarthritis, rhinitis, specific cancers, as well as major depressive disorder (MDD) [3]. These conditions have led to increasing difficulty in providing proper care for obese patients, especially during surgical operations. Specifically, anesthesiologists must take into account an innumerable amount of perioperative risks associated with obese patients.

Currently, in the literature, obesity due to its many co-existing comorbidities serves as a risk factor for increased surgical complications and significantly higher mortality [4,5]. Not only must an anesthesiologist review the health conditions and BMI of the patient, but it is also vital to be aware of the physiological challenges prone in an obese patient, such as respiratory or cardiac changes as well as anatomic variations [6]. Additionally, excess body weight can limit exposure in the operation field and prolong surgery time thus requiring anesthetic care [4]. Finally, appropriate anesthetic drugs and dosages in obese patients must be evaluated.

Despite the growing recognition of these challenges, there are specific gaps in current knowledge that hinder the ability of anesthetic teams to provide optimized care for this patient population. Firstly, there is a lack of consensus on the optimal anesthetic techniques and strategies to minimize perioperative risks and complications in obese individuals. For example, surgical complications that are prevalent in patients with obesity, such as obstructive sleep apnea, warrant comprehensive guidelines for managing these issues. A recent workshop by the American Thoracic Society acknowledges this gap in evidence-based approaches to optimize care of patients with obstructive sleep apnea (OSA) during surgery, citing the need to better classify patients at greatest risk, evaluate current prevention strategies like continuous positive airway pressure (CPAP), and develop better monitoring techniques [7]. Additionally, the impact of obesity on drug pharmacokinetics and pharmacodynamics remains an important knowledge gap. Obese patients often exhibit altered drug distribution, clearance, and response to anesthesia, which can have significant implications for drug dosing and titration. Yet, obese patients are not included in most clinical trials for anesthetic agents, and as a result, there are limited guidelines on the appropriate size descriptors, such as total body weight versus ideal body weight, in pharmacokinetic and pharmacodynamic studies [8]. An assessment of the pharmacological considerations specific to obesity is crucial for achieving optimal anesthesia outcomes and patient safety. Given these knowledge gaps and the increasing prevalence of obesity, it is imperative to conduct a comprehensive review of the existing literature on the anesthetic management of obese patients. By analyzing the current evidence, this review aims to identify areas of consensus, highlight shortcomings of current approaches, and provide recommendations to guide anesthetic teams in delivering optimal care to this high-risk patient population.

Thus to effectively perform surgery on an obese patient, the anesthetic team requires varied and extensive knowledge of the subject and possible complications. In order for the team to be sufficiently prepared to manage any complications that may occur during surgery, it is also extremely important to be able to identify any anesthesia-related risk factors specific to the patient. Therefore, the purpose of this review is to analyze and discuss the common comorbidities and operative variability an anesthesiologist must evaluate while overseeing patients with obesity during the different stages of surgery including pre-operatively, intraoperatively, and postoperatively. The goal is to prepare anesthetic teams with the information necessary to provide proper surgical care to patients with obesity, as well as the requirements of managing an obese patient before and after surgery. Complications can vary among individuals and may not be applicable to every obese patient. Furthermore, this paper will discuss different anesthetic medications, the challenges they present for obese patients, and treatment strategies that minimize complications. Lastly, this paper will discuss the rationale and data of new medication strategies that are being tested clinically to provide a safer anesthetic experience in obese patients.

## Review

Methodology

**Figure 1:**
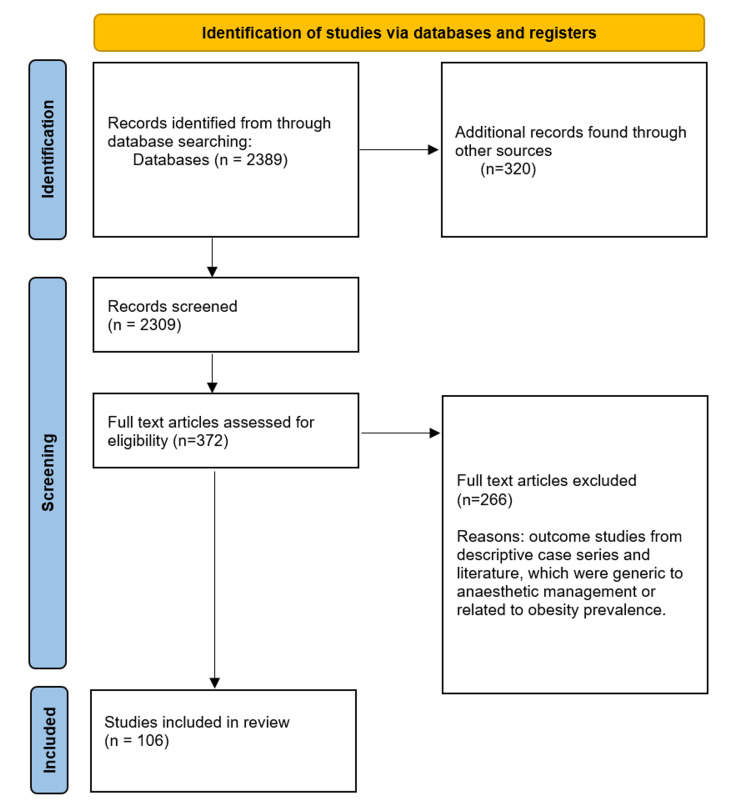
PRISMA Flow Chart for Search Strategy PRISMA: Preferred Reporting Items for Systematic Reviews and Meta-Analyses

A thorough search of multiple databases was conducted, including Google Scholar, Pubmed, and Scopus, in order to identify case reports, case series, clinical studies, and reviews that discussed the epidemiology, clinical presentation, pathogenesis, and histological examination of anesthetic management in obese patients. The search methodology was conducted according to Preferred Reporting Items for Systematic Reviews and Meta-Analyses (PRISMA) guidelines, and the PRISMA flow chart was included below to illustrate the process (Figure 1). The search yielded relevant literature, which was carefully analyzed and synthesized in the results section of this report. Through this process, we gained a better understanding of the challenges and complexities involved in managing anesthesia in obese patients, as well as the various strategies and techniques that can be employed to improve patient outcomes. Specifically, our analysis focused on factors such as the pharmacokinetics of anesthetic agents in obese individuals, the impact of obesity on airway management, and the potential risks and complications associated with anesthesia in this population.

Epidemiology

Obesity is the excessive or abnormal accumulation of fat or adipose tissue in the body [1,2]. It is usually caused by an energy imbalance between the amount of energy taken in from food and drinks, and the amount of energy expended through physical activity and metabolic processes [1,2]. Genetics, endocrine disorders, medications, and psychological factors are other means that can lead to the development of obesity. Furthermore, culture, economic factors, as well as reduced physical activity, insomnia, and the accessibility and consumption of high-fat, high-carbohydrate foods contribute to the development of obesity [1-3]. Endocrine disorders can cause the body to produce too much of certain hormones, like cortisol and insulin, leading to weight gain [3]. Certain medications, like steroids, can also cause weight gain [3]. Psychological factors, such as stress, can also lead to overeating and lack of physical activity, both of which can cause weight gain. Finally, living in an environment with easy access to high-fat, high-carbohydrate foods can lead to obesity [9]. Genetics can influence how the body stores fat and how the body can respond to different diets. Numerous studies have already established the link between genetics and obesity [9]. It has been shown that the FTO gene is related to adiposity [9]. This gene is linked to higher levels of body fat and a greater risk of being overweight and obese. It is estimated that individuals with two copies of the FTO gene variant have a 30% higher risk of obesity. Adipokines are hormones released from fat cells that can cause inflammation and blood clots, increasing the risk of stroke [9]. Additionally, obesity is associated with other health risks such as heart disease, diabetes, and cancer. The link between genetics and obesity is an important factor to consider when looking at overall health [9,10]. Additionally, research has indicated that this gene can be linked to other metabolic diseases, such as type 2 diabetes. Visceral obesity is negatively correlated with adiponectin, a cytokine that reduces food intake and body weight [9-12]. Adipocytes also produce leptin, which reduces food consumption and body weight. Insulin resistance and triglyceride levels increase as a consequence of obesity, contributing to left ventricular dysfunction [10-12].

Categorization of obesity

Body mass index or BMI is a statistical measure that uses height and weight to estimate body fat in males and females taking into account age. BMI is calculated by taking the weight in kilograms (kg) and dividing it by the height in meters squared (m²). Obesity is defined as a BMI greater than or equal to 30 kg/m². It is further divided into Class I, a BMI from 30 to 34.9 kg/m²; Class II, a BMI from 35 to 39.9 kg/m²; and Class III, a BMI greater than or equal to 40 kg/m², at which point it is considered severe obesity [13]. The WHO categorizes people into four categories based on their BMI: underweight, normal, overweight, and obese (Table 1). The terminology was changed from desirable for a BMI up to 25, "grade I obesity" between 25 and 29.9, "grade II obesity" between 30 and 40, and "grade III obesity" greater than 40. In addition, the WHO classification of overweight was based on a BMI of 27.8 for men and 27.3 for women. The National Institute of Health (NIH) cutoff point was reduced to 25 in 1998, instantly converting millions of Americans from being normal weight to being overweight [14].

**Table 1:** WHO Categories of Body Mass Index

Classification	BMI (kg/m²)
Underweight	<18.5
Normal Weight	18.5-24.9
Overweight	25-29.9
Obese	≥ 30
Class I	30.0-34.9
Class II	35.0-39.9
Class III	≥ 40

Morbidity and mortality risk

Obesity, defined as BMI ≥30 kg/m^2^, presents with major comorbidities and complications attributing to an associated increased all-cause mortality risk. A systematic review and meta-analysis by Flegal et al. found the hazard ratios (HR) for all-cause mortality for obesity (BMI ≥30 kg/m^2^) relative to normal weight (BMI 18.5 to <25 kg/m^2^) to be 1.18 (95% CI, 1.12-1.25) [15]. Additionally, the HR for obesity grade 2 (BMI 35 to <40 kg/m^2^) and obesity grade 3 (BMI ≥40 kg/m^2^) was found to be 1.29 which was also associated with a higher all-cause mortality rate. Furthermore, for every 5-unit increase in BMI above 25 kg/m^2^, overall mortality increases by 29%. The increased risk of death is especially apparent from cardiovascular disease (CVD) and cancer, however, the association varies between populations [16,17].

Obesity also presents a plethora of comorbidities and complications in a significant portion of the patient population. Evidence for statistically significant associations for obesity was found with the incidence of type II diabetes, all CVDs, asthma, gallbladder disease, osteoarthritis, chronic back pain, and all cancers except esophageal and prostate cancer [18]. Other comorbidities that have been associated with obesity include obesity-hypoventilation syndrome, chronic kidney disease, non-alcoholic fatty liver disease, female subfertility, and gastroesophageal reflux [7,17]. This highlights the wide range of conditions that are affected by excess body weight and can present as additional considerations for individuals undergoing procedures requiring anesthesia.

Diabetes

“Diabesity” is the term that is used for the overlap between the adverse health effects imparted by obesity and type 2 diabetes mellitus (T2DM) [19]. Currently, the global prevalence of T2DM among adults is 10.5% and is expected to increase to 12.2% by 2045 [20]. The relative risk of the comorbidity of T2DM and obesity is 6.74 among males and 12.41 among females [18]. The pathophysiology that links obesity and T2DM is through the increased number of circulating free fatty acids in obesity-promoting oxidative stress [21]. Specifically, elevations in triglyceride-rich lipoproteins and reductions in high-density lipoprotein-cholesterol are seen in obesity. This dysregulation in turn leads to chronic low-grade inflammation and subsequently peripheral insulin resistance [22].

Cardiovascular Disease (CVD)

Obesity has long been linked to CVDs including hypertension, coronary artery disease, congestive heart failure, and stroke. This is highlighted by the relative risks of obesity and hypertension (1.84 in males vs 2.42 in females), coronary artery disease (1.72 in males vs 3.10 in females), and congestive heart failure (1.79 in males vs 1.78 in females) [18]. In fact, obese children are three times as likely to have hypertension than non-obese children and obese individuals are twice as likely to have a stroke than those with BMI <23 [23].

Cancer

The worldwide burden of cancer attributable to obesity, in terms of population-attributable fraction, is 11.9% in men and 13.1% in females [24]. The relative risk in females for breast cancer, endometrial cancer, and ovarian cancer was 1.13, 3.22, and 1.28 respectively [18]. In both males and females, excess body weight is associated with a significant relative risk for colorectal, kidney, and pancreatic cancer [18]. Additionally, the study found breast cancer to have a 46% greater chance of developing distant metastases in obese individuals and a significantly increased risk of death by 38% [25]. The study by Ewertz et al. concludes that some cancers defer a poorer prognosis in obese individuals [25].

Anatomical airway changes in obese patients

When evaluating the surgical patient, it is imperative that the anesthesiologist and other members of the anesthesiology team conduct a thorough physical examination to assess the airway of the patient. When compared to the non-obese population, studies have demonstrated that obesity is a risk factor for difficult intubation [26]. Due to the anatomical and physiological changes associated with morbid obesity, airway management can be a challenge [27]. Morbid obese patients have an increased lingual fat content and tongue volume [28]. A large tongue can obstruct the view of the larynx, disrupting visualization during intubation and increasing the risk of adverse events [29]. Furthermore, the increase in tongue volume can obstruct the visualization of the posterior facial pillars and soft palate, masking the visibility of the uvula, an additional component of achieving successful intubation [29]. Morbid obesity is also associated with a short muscular neck [27]. A short muscular neck limits mobility and thus proper neck positioning in an obese patient is more difficult comparatively [27]. Upper airway collapse is a factor causing difficult intubation in obese patients due to the additional adipose tissue within the pharyngeal walls [27]. Increased neck circumference has been shown to be a factor in difficult intubation [30]. A study conducted by Brodsky et al. demonstrated that a neck circumference of 60 cm is associated with a 35% probability of difficult intubation compared to a neck circumference of 40 cm having a 5% probability of problematic intubation [30]. A neck circumference ≥35.5 cm in men and ≥32 cm in women is considered the cut-off point for obesity [30,31]. Brodsky et al. concluded that a Mallampati score of III or IV combined with a neck circumference greater than 40 cm is the greatest predictor for problematic intubation [30]. Morbidity and mortality associated with difficult intubation can be greatly reduced by understanding the anatomical airway changes in obese patients.

Furthermore, careful attention to positioning can be helpful in managing the airway of obese patients. For example, the head-elevated laryngoscopy position (HELP) is a technique that involves elevating the head and shoulders of the patient to improve laryngeal visualization during intubation [32]. Likewise, the use of a modified ramp position can help achieve a similar effect in some patients with obesity [33]. Devices like video laryngoscopes can provide a better view of the larynx and vocal cords and may improve intubation success rates in obese patients [34].

A greater understanding of the specific physical intubation challenges in obese patients can help an anesthesiologist better prepare and plan for intubation. Careful preoperative assessment, position, and use of appropriate equipment can help to mitigate these challenges. Awareness of anatomical variations in obese patients and subsequent modification of technique are crucial in ensuring quality patient care and improving patient outcomes in surgery. Airway management in these patients should be clearly communicated with a backup plan established beforehand.

Preoperative care of obese patients and risk prediction

Organization and Equipment

Obese individuals are at increased risk for perioperative complications and thus require increased emphasis on preoperative assessment [35]. This can be attributed to but is not limited to, airway access, peripheral vascular access, presence of comorbidities, and patient positioning. Therefore, it is important to provide proper pre-oxygenation and have alternative airway devices, such as video-assisted laryngoscopes as well as ultrasounds for peripheral access [36]. A study by Moore et al. demonstrated that awake tracheal intubation using video laryngoscopy was successful in 50 obese patients undergoing bariatric surgery using sedation and topical anesthesia [37]. This is imperative because, at increased body weights, the odds of requiring greater than one attempt with direct laryngoscopy were greater when compared to lean patients [38]. Therefore safety can be improved by being familiar with and having proper equipment prior to the commencement of anesthesia in obese patients.

Respiratory Assessment

In patients that are obese (BMI ≥30 kg/m^2^), intra-abdominal pressure is increased while total vital capacity and functional residual capacity are decreased. These changes increase the risk for pulmonary atelectasis in obese patients, especially in the setting of abdominal or cardio-thoracic surgeries [39]. Anesthesiologists should also carry out a standard questionnaire assessing for prior history of difficult intubation or OSA. The STOP-BANG Questionnaire includes snoring, tiredness, observed apnea, high blood pressure, BMI, age, neck circumference, and male gender [40]. A high total score is associated with an increased risk of OSA [40]. In patients who are obese or have a high STOP-BANG score, measures including the use of protective ventilation (pre-, peri-, and postoperative continuous pressure) morphine sparing, and semi-seated positioning are recommended [41]. To fully assess for difficult mask ventilation, patients should be assessed for jaw protrusion, lack of teeth, snoring, Mallampati, the width of mouth opening, sternomental distance, thyromental distance, and neck circumference [42,43]. However, it is important to note that only a high Mallampati score and large neck circumference were predictors of intubation problems [43].

Cardiovascular Assessment

Due to the high prevalence of comorbid CVDs related to obesity, an electrocardiogram (ECG) is recommended to identify undiagnosed abnormalities [44]. This is particularly important because of the functional and structural changes that obesity produces in the heart that can lead to an increased risk of atrial fibrillation (AF) and other arrhythmias [45]. The primary proposed mechanism implicates oxidative stress and inflammation as key mediators in the development of AF [46]. Additionally, every unit increase in BMI increases the risk of AF by 3%, further reinforcing this relationship [47]. Right-axis deviation and right bundle-branch block findings on an ECG suggest pulmonary hypertension while the left-bundle branch is suspicious for coronary heart disease (CHD) [48].

An additional object to monitor for is the presence of comorbid OSA, which causes pulmonary hypertension and autonomic activation contributing to the development of AF [49]. Therefore, proper blood pressure cuffs or thigh cuffs and equipment should be secured to provide an accurate measurement throughout. Severely obese patients also may reasonably warrant an arterial blood gas measurement and chest radiograph [48]. Thus, it is important that obese individuals be assessed for arrhythmias and structural abnormalities in the preoperative period. Perioperative cardiovascular morbidity can be mitigated by being aware of the following risk factors: (1) high-risk surgery, (2) history of CHD, (3) history of congestive heart failure, (4) history of cerebrovascular disease, (5) preoperative insulin treatment, and (6) preoperative creatinine level >2.0 mg/dL [48].

Pre-oxygenation

Pre-oxygenation in obese patients is important due to a greater risk of desaturation and significantly higher oxygen consumption and reduced functional residual capacity [50]. Safe apnea time is reduced which causes a faster time to desaturation than patients with normal BMI [51]. During apnea, the body consumes 250mL/minute of oxygen while producing 200mL/minute of carbon dioxide, resulting in rapidly progressing respiratory acidosis. The continual consumption of oxygen creates a pressure differential in the lungs causing mass movements of gasses from upper airways into the alveoli. The introduction of a high concentration of oxygen at any airway level can be a useful tool to prevent desaturation by increasing alveolar oxygen stores [38]. A study by Patel et al. examined the use of transnasal humidified rapid-insufflation ventilatory exchange (THRIVE) as a physiological method of increasing apnea time in obese patients. THRIVE uses CPAP and gas exchange through flow-dependent dead-space flushing to provide beneficial impacts on apneic oxygenation. They were able to demonstrate a median apnea time of 14 minutes for all patients in the study with no patient having arterial desaturation (SpO_2_ < 90%) [52]. Additionally, the reduced functional residual capacity in obesity leads to decreased oxygen reserve; therefore, oxygenation is recommended in the reverse Trendelenburg position or “ramped-up” position aligning the external auditory meatus to the sternum [49,50]. Safe apnea time has been shown to be further extended via the use of nasal prongs [51]. The oxygen delivery can continue during the apneic phase through nasal cannula use. Other methods of pre-oxygenation include CPAP, a high-flow nasal cannula (HFNC), and non-invasive ventilation (NIV) with the common theme of quality oxygenation to improve the margin of safety in obese patients [51].

Preanesthetic Medication

Proper pre-anesthetic medication relies on individual needs, type of surgery, and availability of anesthetic agents and techniques. Initially developed to counteract the adverse effects of anesthesia, premedication is used to improve general well-being and satisfaction [53]. In patients that are obese, premedication can help to prevent surgical complications, such as post-surgical pain, anxiety, gastrointestinal disturbances, and optimization of underlying medical conditions. In patients that are morbidly obese, there is increased cardiac output, increased lean body weight, increased extracellular fluid volume, and increased fat mass [54]. All of these factors can affect the pharmacodynamics and pharmacokinetic properties of administered medications [54]. Antimicrobials, such as cefazolin, are administered for surgical site prophylaxis. While most guidelines recommend 3g of cefazolin for patients weighing ≥120 kg versus 2g for patients weighing <120 kg, there is no consensus [55]. There are consistent findings that report that cefazolin tissue concentrations are inversely correlated with the BMI of the patient [56]. Despite this, a systematic review by Coates et al. supports that a 2g dose of cefazolin is sufficient in preventing surgical site infections for surgery lasting up to 4 hours [55,56]. Therefore while a 2 g dose of cefazolin may be sufficient, further randomized control trials are needed in the area. Obese patients also have a higher risk of developing hypertension over time [57]. Among gastrointestinal disturbances, the most common is postoperative nausea and vomiting (PONV) in obese patients. It is recommended in high-risk patients to treat with at least a combination of two or three different receptor antagonists, rather than increasing the dose of a single receptor antagonist. The most effectively used agents include ondansetron combined with dexamethasone and consideration of the addition of haloperidol [57,58]. A randomized, controlled, double-blind study conducted by Benevides et al. evaluated the intensity of nausea and pain post-laparoscopic sleeve gastrectomy on 90 obese patients who received either ondansetron alone (Group O), ondansetron plus dexamethasone (Group OD), or ondansetron plus dexamethasone plus haloperidol (Group HDO) [58]. Statistically significant results showed that nausea intensity, pain, and morphine consumption were lower in Group HDO when compared to Group O [58]. Therefore combination therapy in obese patients is much more efficacious. In addition, patients with health problems are known to have an increase in their level of anxiety prior to surgery. For the management of anxiety, generally, benzodiazepines are used along with consideration for clonidine, and alpha-2-adrenergic receptor agonists [53]. Proper pre-anesthetic medication evidenced by recent trials and reviews can improve the overall anesthetic experience for obese patients.

American Society of Anesthesiologists (ASA) Physical Classification System

Determination of the ASA score is based on a comprehensive evaluation of the patient's overall health and surgical risk factors, taking into account their specific medical conditions, including obesity. The ASA score ranges from I to VI, with each class representing different levels of health and surgical risk [59]. Patients who are considered obese with a BMI of 30 to 40 are considered at minimum ASA II and can increase based on other comorbidities or increasing BMI. Obese patients are at risk for many comorbidities as previously discussed, and thus are likely to have increasing ASA classifications. A study by Hackett et al. measured the independent predictive value of the ASA classification system for complications and mortality using multivariate regression [60]. The study used 2,297,629 cases and found increasing levels of ASA to be strongly associated with increased morbidity and mortality [60]. Additionally, the ASA classification system was found to be independently predictive when controlling for comorbidities.

When comparing patients with a high ASA to patients with a low ASA, minor surgeries with lower complexity and physiological impact, pose manageable risks in low ASA obese patients, primarily related to obesity-associated challenges like altered respiratory mechanics and increased cardiovascular strain. For example, a prospective cohort study by Kudsi et al. measured outcomes in robotic inguinal hernia repair in obese versus non-obese patients [61]. The study found no differences in outcomes or other post-operative variables including wound complications, readmissions, or recurrence [61]. Similarly in another study by Tekin et al. laparoscopic anti-reflux surgery was found to be more demanding in obese patients but without any significant increase in complications [62]. On the other hand, major surgeries subject patients to higher physiological stress, presenting elevated risks for both high- and low-ASA obese patients due to procedure complexity and obesity-related challenges. High ASA scores increase surgical risk independently, while obesity further compounds risks by intensifying strain, raising the chance of adverse events, and presenting challenges in anesthesia, access, wound healing, and recovery. Bamgbade et al. performed a retrospective review and analysis of postoperative complications within 30 days of non-cardiac moderate or major surgery [63]. The study showed that total post-operative complications were 7.7%, however, obese patients had a higher prevalence of myocardial infarction, peripheral nerve injury, wound infection, and urinary tract infections [63]. Hence, both minor and major surgeries carry challenges for obese patients with high ASA scores, with major surgeries generally associated with higher risks for all patients, regardless of ASA scores. Using the ASA score, anesthesiologists can stratify patients based on procedural risk and predict outcomes.

Assessment for required postoperative care

The post-operative assessment required in obese patients must take into account the cardiovascular, pulmonary, and skin examinations. Early postoperative complications include deep vein thrombosis, pulmonary embolisms, bleeding, and/or cardiovascular/pulmonary compromise. The cardiovascular assessment begins with a blood pressure measurement which must be taken with an accurately and appropriately sized blood pressure cuff. The pulmonary exam should include listening to the lungs carefully while taking into consideration that the patient may have underlying lung disease, such as asthma, chronic obstructive pulmonary disease (COPD), or obesity hypoventilation syndrome [9,24]. Due to obesity increasing healing time, the skin examination has to focus on wound healing making sure to carefully monitor all incision sites.

Additionally, due to the many comorbidities seen in obese patients, transfer to an intensive care unit (ICU) may be necessitated. Although this should not be routine, but instead be based on many individual characteristics. A multicenter population-based cohort study by Morgan et al. assessed indications, incidence, and outcomes of patients requiring ICU admission after bariatric surgery [64]. The study found that ICU admission post-bariatric surgery was uncommon (4.9%), and a large amount was unplanned (30.9%). Additionally, those that were admitted to the ICU were older, and more likely to be male with the strongest preoperative factors associated being diabetes mellitus, chronic respiratory disease, and obstructive apnea [64]. An additional study by Nofal et al. proposes a scoring system to stratify risk for postoperative ICU admission in obese patients undergoing bariatric surgery [65]. The score includes the following factors significantly related to ICU admission: older age, male gender, higher BMI, higher ASA, OSA, and spirometry results [65]. Therefore, due to the unplanned nature of ICU admission in obese patients due to the presence of comorbidities, we recommend they be stratified for potential ICU admission as part of preparation.

Intraoperative care

Intraoperative care is highly important in the management of obese patients. To start, the positioning of the patient plays a large role in successful procedures. The patient must be comfortable and safe while also maintaining efficiency for the surgical team completing the operation. The most well-tolerated position for obese patients is the lateral position [66]. In the lateral decubitus position, respirations and ventilation are easier than in the supine position. In the prone position, there is compression of the aorta and inferior vena cava, leading to poor perfusion. The lateral decubitus position allows for better perfusion. The operating room staff must be careful to use appropriate techniques when lifting, positioning, and transporting the patient to ensure safety on both sides of the spectrum [66]. Additionally, to improve operating room safety, proper tables must be available to prevent the increased risk of falling off due to instability and weight load shifts [66]. A cross-sectional study by Balci et al. determined the risk of intraoperative pressure injuries in patients undergoing elective cranial surgery which had an average duration of 5.5 hours [67]. It was found that surgically obese patients had a higher risk of high-pressure injuries secondary to static friction forces in part due to more fat mass compressing on blood vessels [67]. The BMI index was found to be an independent risk factor for pressure injury development. Thus intraoperatively, when compared to a non-obese patient, those with a higher BMI must be given specific precautions such as additional padding to minimize these outcomes [66].

Flexible fiberoptic laryngoscopy is the most common way to approach awake tracheal intubation. In obese patients, intubation can prove to be difficult because of fat deposits narrowing the airway and decreasing visualization of the vocal cords [68].

Intraoperative monitoring of the obese patient can provide various challenges. Aside from proper positioning and airway maintenance, it is key to monitor ventilation and the effects of anesthesia. Many non-invasive methods have been used to track the patient’s vitals such as blood pressure; however, the most accurate has been an invasive arterial line which depicts blood pressure reliably if placed correctly. Goal-directed fluid therapy (GDFT) helps to measure and optimize tissue perfusion and guide how much fluid resuscitation the patient will need in the postoperative period to avoid complications or worsen pre-existing conditions [69].

Hypnotics

Hypnotics play an important role in the induction and maintenance of anesthesia. While many of the hypnotics discussed in this section are standard practice for both obese and non-obese patients, the altered pharmacokinetics and pharmacodynamics in obese patients affect the recovery times and safety profile of these drugs [50]. To account for these physiological differences, clinicians must therefore adjust the dosage and avoid certain anesthetic agents to best address the unique needs of obese patients.

Propofol is the most commonly used hypnotic agent in obese patients due to its rapid onset of action and quick recovery time. However, being highly lipophilic, the volume of distribution and clearance increases linearly with body weight. Clearance and volume of distribution are considerably higher in obese patients, which can translate to prolonged sedation and longer recovery time [70]. To avoid overdose and adverse effects like hypotension, dosing of propofol in obese patients has been recommended to follow lean body weight (LBW) instead of total body weight (TBW) [69]. One clinical trial of 40 morbidly obese patients reported the optimal dosage to be 2.310-3.567 mg/kg using LBW at which the depth of intubation is satisfactory and hemodynamics are stable in this population [71].

Opioids can be used as an option, but their safety profile is suboptimal in obese patients due to an increased risk of OSA compared to other hypnotic alternatives [72]. Fentanyl is the most commonly used opioid in anesthesia. It has a high volume of distribution and clearance owing to its high lipophilicity. Alfentanil is less lipophilic and less potent than fentanyl, but nonetheless, it is expected to have a higher volume of distribution in obese patients. Furthermore, the therapeutic ability of opioids as anesthetics is difficult to ascertain for obese patients since very few pharmacokinetics/pharmacodynamics studies have been done in this population [73]. Therefore, opioids are generally not recommended for obese patients.

Among inhalation agents, desflurane, isoflurane, and sevoflurane have commonly used sedatives in the bariatric population. However, some studies have shown desflurane to cause airway irritation and coughing, which may serve as a contraindication for patients with OSA [66]. Among these, desflurane is the least lipophilic and thus, it is most promising because of its rapid onset and short half-life. This is of higher importance in obese patients to enable a quicker reversal of anesthesia compared to other chemically similar agents like isoflurane and sevoflurane [74].

Dexmedetomidine is a newer hypnotic agent that has been used in the anesthetic management of obese patients. Dexmedetomidine has a unique mechanism of action that selectively targets the α2-adrenoceptor, resulting in sedation, anxiolysis, and analgesia [75]. It has been suggested that dexmedetomidine may have some advantages over other hypnotics in obese patients due to its minimal effect on respiratory function, as well as its ability to attenuate sympathetic responses to intubation and surgery. For example, a randomized clinical trial comparing dexmedetomidine and propofol in patients undergoing bariatric surgery showed that dexmedetomidine was associated with less PONV and better pain control compared to propofol [76]. In laparoscopic bariatric surgery, a loading dose of 0.5 mg kg-1 h-1 given over 10 minutes with an infusion rate of 0.2 mg kg-1 h-1 has been recommended [73].

Dosage requirements of drugs for patients that are morbidly obese are generally not scaled off TBW as would be the case for normal-weight individuals [73]. In fact, dosing based solely on TBW can result in an overdose in this patient population. Ingrande et al. instead recommend other metrics such as ideal body weight (IBW), body surface area (BSA), BMI, and LBW. Specifically, LBW is the difference between TBW and fat mass and generally increases with increasing TBW [73]. The authors of the study conclude that induction doses in morbidly obese patients for hypnotics such as thiopental, propofol, and etomidate should be thus based on LBW instead [73].

Neuromuscular blockers

Neuromuscular blocking agents (NMBAs) are administered via endotracheal intubation during anesthesia to help improve surgical outcomes. NMBAs act to reduce the risk of vocal injuries as a result of intubation and can provide benefits to patients with low lung compliance [77]. Meta-analysis of 34 clinical trials has shown that avoiding NMBAs during tracheal intubation led to significantly high rates of difficult intubation and discomfort [78].

Depolarizing neuromuscular blocking agents (eg. succinylcholine) and nondepolarizing neuromuscular blocking agents (eg. rocuronium, vecuronium, atracurium) are the two main types of NMBAs [77]. Dosage requirements for depolarizing and nondepolarizing agents are listed in Table 2 [79]. These guidelines help anesthesiologists and healthcare providers determine the appropriate administration of NMBAs, ensuring optimal muscle relaxation while minimizing the potential for adverse effects. NMBAs are valuable tools in anesthesia practice, particularly during endotracheal intubation, to mitigate the risk of vocal injuries and aid patients with low lung compliance. Depolarizing and non-depolarizing agents offer distinct mechanisms of action and are administered based on specific clinical requirements. Understanding the appropriate dosage and usage of NMBAs is essential for anesthesia providers to promote patient safety and achieve favorable surgical outcomes.

**Table 2:** Dosage Requirements of Neuromuscular Blockers for Obese and Non-Obese Patients

Drug	Intubation Dose (mg kg−1)
Succinylcholine	1.0
Rocuronium	0.6
Vecuronium	0.1
Atracurium	0.5

NMBAs play a crucial role in anesthesia procedures, particularly during endotracheal intubation, by enhancing surgical outcomes. These agents are employed to minimize the risk of vocal injuries associated with intubation and offer potential benefits to patients with low lung compliance such as those that are obese [77]. NMBAs are categorized into two main types: depolarizing neuromuscular blocking agents, such as succinylcholine, and non-depolarizing neuromuscular blocking agents, including rocuronium, vecuronium, and atracurium [77]. Endotracheal intubation, a common procedure performed under general anesthesia, involves the placement of a flexible tube into the trachea to maintain an open airway during surgery. While it is a vital technique, intubation can potentially cause damage to the vocal cords and surrounding structures. NMBAs are utilized to facilitate intubation by temporarily paralyzing the skeletal muscles, including the vocal cord muscles. This paralysis helps prevent involuntary movements and spasms of the vocal cords, reducing the risk of trauma or injury during intubation [77].

In addition to their role in preventing vocal injuries, NMBAs can also be advantageous for patients with low lung compliance. Lung compliance refers to the ability of the lungs to expand and accommodate changes in pressure during the breathing cycle. Conditions such as acute respiratory distress syndrome (ARDS) and pulmonary edema can lead to decreased lung compliance, making it more challenging for the patient to breathe effectively. By inducing muscle relaxation, NMBAs reduce the work of breathing, enhance lung mechanics, and optimize ventilation in patients with compromised lung compliance [77]. There are two primary categories of NMBAs: depolarizing and non-depolarizing agents. Depolarizing agents, exemplified by succinylcholine, work by initially stimulating the nicotinic acetylcholine receptors (nAChRs) at the neuromuscular junction. This stimulation leads to a depolarization phase, causing muscle contraction. However, succinylcholine then persists at the receptor site, preventing the muscle from repolarizing and ultimately resulting in muscle paralysis. Non-depolarizing agents, on the other hand, competitively bind to nAChRs, blocking the action of acetylcholine and preventing muscle contraction. The non-depolarizing NMBAs, such as rocuronium, vecuronium, and atracurium, differ in their potency, duration of action, and side effect profiles [77].

Succinylcholine is the drug of choice for depolarizing NMBAs. It is primarily used for short procedures (<30 min) due to its rapid onset and short duration of action [77]. The dose of succinylcholine required for endotracheal intubation is 1.0 mg kg^-1^ [79]. Succinylcholine’s mechanism of action binds to postsynaptic cholinergic receptors on the motor end plate, resulting in rapid depolarization, fasciculation, and flaccid paralysis. However, it is important to note if the patient has functional plasma pseudocholinesterase, as this enzyme is metabolized succinylcholine. Thus, without pseudocholinesterase, the patient has a prolonged reaction to succinylcholine, which can require postoperative mechanical ventilation [80].

There are two types of non-depolarizing NMBAs, steroidal (e.g. rocuronium, vecuronium, pancuronium) and benzylisoquinoline (e.g. atracurium, mivacurium, cisatracurium). Non-depolarizing NMBAs work by competitively inhibiting acetylcholine on postsynaptic nicotinic receptors, producing longer muscle paralysis as compared to depolarizing NMBAs [81].

The adverse effects of succinylcholine may warrant its exclusion for some patients. It should be avoided in patients with hyperkalemia, severe burns, denervating disease, and a history of malignant hyperthermia [82]. Whereas, non-depolarizing NMBAs have been linked to increased histamine release and may create hemodynamic instability [83].

Monitoring of NMBAs is important in the management and prevention of adverse effects. It is done through train-of-four (TOF) stimulation, which uses electrical stimulation to a chosen muscle group and measures the respective number of twitches [84]. As paralysis increases, twitches in the train decrease in amplitude [85]. Over time, there is a progressive disappearance of the fourth, third, second, and first twitch [85]. The train-of-four ratio is determined by the amplitude of the fourth twitch divided by the first twitch [85]. A decreasing ratio indicates a stronger degree of paralysis [85]. A train-of-four count (TOFC) score of 1-4 is assigned depending on the percentage of receptors blocked; 1: >95%, 2: 85-90%, 3: 80-85%, 4:70-75%. When the TOFR score is <0.9, it can be an alarm for residual NMBA in the patient and often requires the use of a reversal agent [84].

A case-control study by Sadleir et al. showed that obesity was an independent risk factor for NMBA anaphylaxis [84]. One hundred five patients were recruited with an ASA status I or II [84]. Patients were randomly assigned to one of four groups: TOF, double burst stimulation 3, 3 (DBS3,3), double burst stimulation 3, 2 (DBS3,2), or videography [84]. Patients were recruited based on having no neuromuscular, hepatic, or renal disorders [84]. In addition, patients were not taking any drugs that affected the action of relaxants [84]. The percentage of detection of fade in response to the study groups (TOF, DBS3, 3, and DBS3, 2) at the thumb and index finger was recorded and compared using chi-squared tests [84]. In addition, analysis of variance and Scheffe’s multiple comparisons were used to analyze the distances of the movements of the tip of the thumb and index finger [84]. The odds ratio for obese (>29.9 kg/m^2^) patients for NMBA anaphylaxis was 3.8 times greater for obese patients and 7.0 times greater for morbid obesity (>40kg/m^2^) [86]. The authors concluded that this demonstrates a positive relationship between BMI and the risk of adverse reactions to NMBA administration.

One approach to minimizing the risk of adverse reactions to NMBAs in obese patients is to use adjusted dosing. Dosage requirements for NMBAs are based on IBW, which may underestimate the dose required in obese patients. Therefore, it is recommended to use adjusted dosing based on total body weight or lean body weight [87]. For example, a study by Sakızcı-Uyar et al. found that adjusting rocuronium dosing based on corrected body weight resulted in longer neuromuscular blockade duration compared to adjusting based on LBW, underscoring the importance of individualized dosing to achieve adequate muscle relaxation while minimizing the risk of adverse reactions [88]. Additionally, current guidelines by the Society of Critical Care Medicine suggest that IBW or adjusted body weight, not actual body weight, should be used to calculate doses of non-depolarizing NMBAs in obese patients [87]. However, there is limited clinical trial data to fully support this, as none of the clinical trials commented on in the Erstad et al. paper evaluated doses or size descriptors of NMBAs as sustained infusions in normal-weight versus obese populations [8,87].

Reversal of neuromuscular blocking agents

There are two main types of drugs that reverse neuromuscular blockade agents. Acetylcholinesterase is used after the patient has already begun to recover from a neuromuscular block. Neostigmine is a common acetylcholinesterase used for reversing neuromuscular blockades by allowing for acetylcholine to remain within the neuromuscular junction. Neostigmine takes at least 8 minutes before its effects are maximal in patients. An increase in the dosage of neostigmine may not increase its effects [89]. Half of plasma Neostigmine is dependent on renal excretion to get cleared from the body. Therefore, neostigmine is contraindicated for patients with renal failure due to its increased muscarinic effects like bradycardia [90].

Sugammadex is a selective relaxant binding agent that is used to reverse neuromuscular blocks. Sugammadex has a negative lipophilic core that irreversibly binds strongly to steroidal muscle relaxants, rocuronium or vecuronium (NMBAs). It then is subsequently excreted unchanged by the kidneys. Sugammadex can fully relieve a neuromuscular block based on its dose and is more effective at clearing neuromuscular blocks than neostigmine. Sugammadex has more severe risks including rare cases of anaphylaxis and possible vagal bradycardia in patients with pre-existing cardiac problems [89,91]. The American Society of Anesthesiologists Task Force on Neuromuscular Blockade recommends Sugammadex over neostigmine for reversal of rocuronium or vecuronium from a deep to the shallow depth of a neuromuscular block due to sugammadex providing less residual neuromuscular block and a faster recovery period [85]. Dosage should be based on actual body weight, although lower doses have been used successfully in morbidly obese patients [91].

A systematic review and meta-analysis by Subramani et al. determined the efficacy and safety of sugammadex versus neostigmine in reversing neuromuscular blockade in morbidly obese patients [92]. The primary objective was the measurement of recovery time from drug administration from moderate or deep blockade. The results reported that sugammadex significantly reduced the mean time of reversal versus neostigmine for moderate neuromuscular blockade; reversal time was 2.5 minutes and 18.2 minutes, respectively [92]. Additionally, there were fewer adverse effects reports with sugammadex (21.2% of patients) versus neostigmine (52.5% of patients) [92]. Thus, for patients that are obese and undergoing surgery, sugammadex should be considered over other forms of neuromuscular blockade reversal.

Postoperative care

Postoperative care is important for all patients; however, physicians and the entire care team should be cognizant of potential complications that can arise in patients who are obese. Mentioned below is a summary of the potential complications that can arise in the postoperative period for obese individuals.

Patients who have obesity-related comorbidities should be monitored closely in the postoperative period. In the hours post-surgery, there is a decrease in tidal volume by 20% and a 20% increase in respiratory rates, leading to the potential of diaphragmatic dysfunction [9]. Atelectasis has been shown to be more common in morbidly obese patients when compared to nonobese patients [24]. Although resorption occurs within 24 hours in nonobese patients, concerns for atelectasis can persist longer in obese patients [24]. Atelectasis formation is increased in obese patients due to decreased functional residual capacity, reduction in lung volume, and high intraabdominal pressure [93]. Furthermore, a nonobese patient is more likely to be mobilized soon after surgery compared to a morbidly obese patient [93]. Lack of immobilization after surgery contributes to the development of atelectasis after tracheal extubation [93]. To decrease the risk of atelectasis, CPAP can be used in patients after extubation [9]. The development of using CPAP has been shown to improve airway patency and respiratory mechanics, preventing alveolar collapse [9]. In addition, noninvasive positive pressure ventilation (NPPV) can be used to decrease the likelihood of acute respiratory failure, decreasing the risk of developing atelectasis and potentially pneumonia [9].

Perioperative venous thromboembolism (VTE) is a potential complication of any individual undergoing surgery; however, obesity has been shown to be a risk factor for the increased likelihood of developing VTE. VTE prophylaxis, such as sequential compression devices and chemoprophylaxis, should be utilized in the postoperative period to decrease the potential of developing VTE [93].

Obesity can increase the likelihood of having post-surgical wound infection and dehiscence [93,94]. Due to hypoperfusion, impaired inflammatory mechanisms, and decreased delivery of antibiotics in obese surgical patients, wound infections are more likely to occur. Dehiscence can develop due to increased tension on the wound edges caused by an increased body habitus [93]. It is also important to note that skin folds create an environment for bacteria to live, leading to an increase in developing infection and tissue breakdown. The innate immune system is significantly impaired in obese patients due to the damaged peripheral blood mononuclear cells [93].

Cardiovascular impairment can be seen in morbidly obese patients [95]. Because of this, hemodynamic monitoring should be used in the postoperative period to allow for correct fluid replacement. In addition, hemodynamic monitoring can assess cardiac performance, decreasing cardiovascular compromise in obese surgical patients [95]. Cardiac index and left ventricular stroke work remain depressed in the postoperative period in the obese patient. The stress of surgery in an obese patient has been shown to cause left ventricular dysfunction [15,95].

Recent advancements

Conventional general anesthetic opioid treatment has been shown to increase the risk of preventable respiratory distress in obese patients [96,97]. New clinical trials are exploring treating obese patients with opioid-free regimens by reducing the amount and dosage of opioids in the treatment modality with the goal of decreasing the incidence of preventable respiratory complications. Two drugs of note are ketamine (NMDA receptor antagonist) and dexmedetomidine (hypnotic).

Ketamine is an old FDA-approved drug for anesthetic in surgical procedures and is being used off-label for pain management. The drug has regained popularity as it is an alternative to opioids, new protocols to limit abuse potential and new protocols have been implemented to limit adverse effects (administer benzodiazepine to reverse any emergence of delirium side effects) [98]. Therefore, current research on ketamine use in obese patients is focused on determining if ketamine reduces the risk of respiratory complications in obese patients and provides adequate pain control during and after surgery. Several clinical trials are showing promising results when ketamine replaces opioids by reducing the risk of respiratory distress in obese patients while still maintaining adequate pain relief [99,100].

A second drug, dexmedetomidine, is also being used as an opioid alternative for obese patients, as it reduces delirium and does not negatively affect respiratory function in patients [101]. Dexmedetomidine has some withdrawal symptoms that may be prolonged with opioid administration [102]. New research is required to study the withdrawal symptoms of the drug and compare them with opioid addiction to see if its abuse potential is higher. Several clinical trials show that dexmedetomidine decreases the risk of respiratory distress with lower opioid dosage and adequate pain reduction [103,104]. Dexmedetomidine is approved for use in obese bariatric surgery and for sleep apnea patients as there is sufficient clinical evidence that it is an effective treatment for those procedures [101]. However, not all clinical trials are positive for the drug. A separate randomized control trial compared an opioid-free anesthesia method using dexmedetomidine versus a general anesthesia regimen using remifentanil and morphine (opioids) postoperatively for major and intermediate non-cardiac surgeries. The trial was halted due to a greater incidence of serious adverse events for the dexmedetomidine group including hypoxemia, bradycardia, prolonged post-anesthesia care unit stay, and prolonged extubation time [105]. In this trial, the dexmedetomidine group had worse outcomes. It is not clear if dexmedetomidine will be viable as a treatment option for other types of surgeries.

However, due to the relative dangers of opioid use in obese patients mentioned before, opioid-free regimens can be used for certain surgeries. In a prospective double-blind control study, obese patients were set to receive opioid-based or opioid-free regimens prior to laparoscopic urological procedures [106]. The opioid-free regimen consisted of propofol, dexmedetomidine, lignocaine, and ketamine. The primary outcomes measured were respiratory depression, mean analgesic consumption, and time to rescue analgesia. The results showed that the opioid-free group had significantly less incidence of respiratory depression, less analgesic consumption, and decreased discharge time [106]. Therefore, opioid-free regimens do have promise in certain surgical conditions.

Further research is required for both ketamine and dexmedetomidine to create guidelines, as current research has small patient populations, different dosages, and different monitoring criteria making it difficult to generalize findings. The limitation of small clinical trials reduces the likelihood that severe adverse effects will be adequately noted.

## Conclusions

This review article provides a comprehensive overview of the anesthetic management of obese patients during surgery. It covers preoperative assessment, intraoperative management, postoperative care, and recent advancements in anesthetics for this population. With the increasing prevalence of obesity, healthcare providers need to be knowledgeable about the unique challenges and implications of administering anesthesia to obese patients. The article emphasizes the importance of evaluating obesity-related comorbidities during preoperative assessment and provides recommendations for optimizing anesthetic management. It also discusses considerations for altered pharmacokinetics and drug interactions during surgery and highlights specialized techniques to mitigate side effects. Postoperative care includes monitoring for respiratory and cardiovascular complications and implementing measures to minimize risks. The article explores recent advancements in anesthetic use, particularly opioid-free alternatives such as ketamine and dexmedetomidine, which show the potential to be safer for administration in obese patients due to more favorable respiratory physiology. Overall, this review article serves as a valuable resource to enhance patient safety and improve outcomes in the growing population of obese patients undergoing surgical procedures.
